# mSphere of Influence: Stromal Regulation of Epithelium Containing Viral Oncogenes

**DOI:** 10.1128/mSphere.00467-19

**Published:** 2019-07-17

**Authors:** Jason Bodily

**Affiliations:** a Department of Microbiology and Immunology, LSU Health Sciences Center—Shreveport, Shreveport, Louisiana, USA

**Keywords:** fibroblasts, keratinocytes, organotypic culture, papillomavirus, retinoblastoma, stroma

## Abstract

Jason Bodily works in the field of tumor virology. In this mSphere of Influence article, he reflects on how “Inactivation of Rb in stromal fibroblasts promotes epithelial cell invasion” by Adam Pickard et al. (EMBO J 31:3092–3103, 2012, https://doi.org/10.1038/emboj.2012.153) has impacted his work by making him think about the role of stromal cells in human papillomavirus infections.

## COMMENTARY

My training in tumor virology has focused how human papillomaviruses (HPVs) coordinate the virus life cycle in a differentiating epithelium. At various times, I have focused on different elements of HPV, including viral promoters and viral oncogenes, but the role of the tissue environment has always been a critical consideration. Proper growth conditions for HPV-containing keratinocytes in culture are essential to maintain their ability to differentiate and maintain viral genomes. Because HPV is strictly dependent on keratinocyte differentiation to sustain its life cycle, I have often used organotypic raft cultures as a model system to create a fully stratified, three-dimensional epithelium *in vitro* to support HPV life cycle events. Both raft cultures and traditional keratinocyte cultures include fibroblasts as an essential element: as feeder cells in monolayer culture and as dermal support cells in raft cultures. Thus, as I grew up in science, fibroblasts were always an important element in my work but were there largely as part of the furniture, playing some important, but ill-defined, role in the keratinocyte-centered processes in which I was most interested. Despite the growing understanding of the role of stromal cells in cancer biology (1, 2), what role might be played by fibroblasts in my own system only occasionally crossed my mind.

In the paper “Inactivation of Rb in stromal fibroblasts promotes epithelial cell invasion,” the McCance laboratory reported observations that significantly changed the way that I think about the tissue stroma in the HPV life cycle (3). The authors began with the observations of others that Rb, the famous tumor suppressor, is frequently inactivated in tumor stroma and not just in the tumor cells themselves. Pickard et al. made a similar observation about oropharyngeal cancers, showing that Rb is phosphorylated (inactivated) in the stromata of these tumors. Then they modeled the consequences of this inactivation by creating organotypic raft cultures in which Rb was knocked down in the fibroblast compartment. HPV16 E6/E7-immortalized keratinocytes were used in the epithelial compartment. Strikingly, in the rafts containing Rb knockdown fibroblasts, the keratinocytes became significantly more invasive, growing in clusters down into the collagen layer. The authors then went on to identify keratinocyte growth factor as driving the proinvasive effect of stromal Rb knockdown. The authors drew the conclusion that Rb-mediated pathways in the stroma can regulate the invasiveness of epithelial cells containing HPV oncogenes.

From this paper, I gained three insights that have been important to me. The first was that the stroma, which had always been silently present in my experiments, was likely to be keenly important to the life cycle of HPV. Although Pickard et al. did not study the role of stromal Rb in productive HPV infection, it was clear from their findings that HPV was almost certain to respond to signals from the stroma in the course of its infectious cycle. Second, Pickard et al. showed that a model system with which I was already thoroughly familiar could be a powerful tool to dissect the specific molecular mechanisms involved. Those two insights have grown together to become the primary focus of the funded research in my lab, which is how epithelial-stromal interactions impact the life cycle of HPV.

A third insight goes a little deeper. Many tumor virologists slip into the trap of thinking about their viruses as carcinogens rather than as organisms in their own right. Sometimes we assume that tumor viruses (and even nonviral cancers) target cellular tumor suppressor genes (TSGs) in order to promote cell proliferation. There is a good argument that the virus needs the resources found in a proliferating cell in order to replicate its genome. However, this paper reminds us that TSGs are not simply cell cycle regulators. There is a growing understanding that TSGs and oncogenes have non-cell-autonomous effects that can impact the cellular microenvironment in a multitude of ways (4–8). Many of these effects govern not just cell cycling but immune responses and other conditions that impact viruses as viruses. From an evolutionary perspective, it seems likely that the potentially dangerous oncogenic activities of a tumor virus facilitate propagation of the virus not just through proliferation of the cell but through regulation of the tissue microenvironment. This possibility opens up a wide range of interesting research directions that I look forward to exploring more deeply in the future.
